# Research on Corporate Social Responsibility Coordination of Three-Tier Supply Chain Based on Stochastic Differential Game

**DOI:** 10.3389/fpsyg.2022.783998

**Published:** 2022-02-07

**Authors:** Mingge Yang, Zhuo Yang, Ying Li, Xiaozhen Liang

**Affiliations:** School of Management, Shanghai University, Shanghai, China

**Keywords:** three-tier supply chain, CSR coordination, stochastic differential game, alliance mechanism, cost subsidy

## Abstract

In the Internet era, consumers prefer products with the attributes of social responsibility. Supply chain enterprises strengthen corporate social responsibility (CSR) management for their own development. To improve CSR throughout the supply chain, it requires coordination and cooperation among the members of the supply chain. In this paper, we consider a three-tier supply chain system consisting of a supplier, a manufacturer, and a retailer and use stochastic differential game to study the CSR coordination of the supply chain. The following indicators are investigated under four decision situations, such as the optimal level of CSR effort for the supply chain members, the optimal value of profit for the supply chain members and the supply chain system, and the expectation and variance of CSR goodwill. Some important results are obtained. (i) Compared with decentralized decision-making, the optimal level of CSR effort increases for the supplier and the manufacturer under local alliance decision-making without cost sharing, whereas the optimal level of CSR effort remains unchanged for the retailer. (ii) Compared with local alliance decision-making without cost sharing, the optimal level of CSR effort remains unchanged for the supplier and the manufacturer under local alliance decision-making with cost sharing. When the sum of the marginal profit for the supplier and the manufacturer is greater than half of the marginal profit for the retailer, the optimal level of CSR effort increases for the retailer. (iii) Compared with local alliance decision-making with cost sharing, the optimal level of CSR effort increases for the supply chain members under overall alliance decision-making. (iv) From decentralized decision-making to local alliance decision-making without cost sharing, to local alliance decision-making with cost sharing, and then to overall alliance decision-making, the optimal value of profit increases for the supply chain members and the supply chain system. Also, the expectation and variance of CSR goodwill increase.

## Introduction

In the Internet era, consumers can choose their own shopping methods more rationally. Consumers will not passively accept the products or services provided by suppliers. Instead, they will take the initiative to find suitable merchants through the Internet. In recent years, traditional supply chain enterprises focused on maximizing profit and neglected the performance of corporate social responsibility (CSR), which led to frequent occurrence of quality issues, environmental issues, and labor rights issues. Therefore, consumers pay more and more attention to CSR in the process of the consumption and prefer products with the attributes of social responsibility. The fulfillment of social responsibility by supply chain enterprises can increase the purchase of consumers, which in turn has a positive impact on corporate reputation. Based on this background, we study the coordination and management of supply chain CSR. Our results provide effective suggestions for enterprises to achieve sustainable development.

Some literatures (Tang et al., 2018; Dal Mas et al., 2021; Endrikat et al., 2021) have studied CSR from the perspective of the coordinated relationship between corporate leaders, employees, and other internal members and CSR. But in the process of supply chain CSR management, the self-interested behaviors of the supply chain members can lead to conflict of interests. To improve the CSR management level of the entire supply chain, the coordination and cooperation of supply chain members are required. Therefore, it is of great practical significance for us to study CSR coordination of supply chain companies from the perspective of the supply chain.

Many scholars (Modak et al., 2014, 2015, 2019; Panda and Modak, 2016; Nematollahi et al., 2017; Panda et al., 2017; Modak and Kelle, 2019; Sana, 2020; Wang et al., 2020) have studied the supply chain CSR coordination from the perspective of the two-tier supply chain. However, the supply chain CSR coordination involves more participants in real life. As a springboard for the extension of the two-tier supply chain to multiple levels, the three-tier supply chain is a research focus of CSR coordination in the supply chain. Therefore, some scholars have studied CSR coordination from the perspective of three-tier supply chain. For example, based on a three-tier supply chain composed of the government, the manufacturer, and a retailer with CSR, Jiang et al. (2021) explored the impact of government carbon subsidies and CSR on the supply chain emission reduction. Yao et al. (2021) analyzed the impact of government’s recycling subsidy mechanism and retailers’ CSR investment on waste recycling supply chain, which is composed of a manufacturer, a retailer, and a third-party recycler. Considering the impact of CSR on random market demand, Hosseini-Motlagh et al. (2019) constructed a three-tier supply chain composed of a manufacturer, a distributor, and a retailer and designed a two-tier wholesale price contract to coordinate the supply chain. Panda et al. (2015) studied the supply chain coordination of the “manufacturer–distributor–retailer” in social responsibility environment and proposed a contract negotiation procedure involving two wholesale price discounts and two Nash bargaining products, which can be used to coordinate channels and distribute residual profit. Mondal and Giri (2021) developed a centralized model and three decentralized models to explore the impact of CSR on manufacturers, retailers, and third-party collectors collecting second-hand products in reverse channels.

The literature (Panda et al., 2015; Hosseini-Motlagh et al., 2019; Jiang et al., 2021; Mondal and Giri, 2021; Yao et al., 2021) studied CSR coordination of different responsible entities in three-tier supply chain, but the existing literature did not study CSR coordination of the supplier, the manufacturer, and the retailer in three-tier supply chain. In fact, with the increasing consumer interest in CSR, not only manufacturers are required to invest more during production, but also suppliers are required to strengthen CSR management at source. At the same time, retailers are required to actively promote CSR products to improve CSR goodwill. Eventually, the potential value of CSR products can be realized collaboratively. For example, Huawei incorporates CSR standards into the entire procurement process and supplier life cycle management. It solves CSR issues through industry collaboration and innovative thinking and achieves sustainable development of the supply chain. Furthermore, the above literature did not consider the influence of alliance mechanism on decision-making of the supply chain members. In fact, in the production and operation of products, if an effective profit distribution agreement is reached, the supply chain companies are likely to form alliances, such as two-by-two alliance or three-part alliance. For example, Japan’s automobile and electronics industry has an influential organizational structure called Jinglianhui, which is a typical supply chain emphasizing alliance relationship. Enterprises in Jinglianhui form close alliance to influence decision-making of the supply chain members and improve the performance of the supply chain (Feng et al., 2012). Therefore, we consider the influence of alliance mechanism on decision-making of the supply chain members and study CSR coordination of the supplier, the manufacturer, and the retailer.

As for the CSR coordination of the three-tier supply chain, the existing literatures are all studied from a static perspective, such as the literature (Panda et al., 2015; Hosseini-Motlagh et al., 2019; Jiang et al., 2021; Mondal and Giri, 2021; Yao et al., 2021). In reality, supply chain CSR management is a dynamic process and needs to be studied from a dynamic perspective. Some scholars have studied CSR management of the secondary supply chain from a dynamic perspective. For example, literature (He et al., 2020; Li, 2020; Cheng and Ding, 2021) used differential game to dynamically study CSR management of the two-tier supply chain. However, there is no dynamic research on CSR management of the three-tier supply chain in the existing literature. As a matter of fact, differential game is an important dynamic game, which has made rich achievements in the fields of supply chain enterprises’ production and operation, quality control, advertising, and promotion. Furthermore, the differential game models in the literature (He et al., 2020; Li, 2020; Cheng and Ding, 2021) did not consider the random interferences faced by the system, whereas in reality, the decision-making process is inevitably affected by various random interferences. Therefore, considering the random interferences faced by the system, we construct a stochastic differential game model and study CSR coordination of the supplier, the manufacturer, and the retailer in three-tier supply chain.

In this paper, we introduce stochastic differential game into CSR coordination of the three-tier supply chain. We give Table 1 to intuitively show contributions of this paper and differences between this paper and the previous literatures. (i) The CSR coordination of suppliers, manufacturers, and retailers in the three-tier supply chain is studied in this paper, whereas it was not studied in the previous literatures. (ii) The dynamic optimization is used for three-tier supply chain in this paper, whereas it is used for two-tier supply chain in the previous literatures. (iii) The random interference faced by the system is considered in this paper, whereas it was not considered in the previous literatures.

**TABLE 1:** Summary of relevant literatures.

References	Supply chain structure		Coordination of S, M, R	Alliance mechanism	Model type		Random interference
	two-tier	three-tier			static	dynamic	
Modak et al., 2014	√				√		
Modak et al., 2015	√				√		
Modak et al., 2019	√			√	√		
Modak and Kelle, 2019	√				√		
Nematollahi et al., 2017	√				√		
Panda et al., 2017	√				√		
Panda and Modak, 2016	√				√		
Sana, 2020	√				√		
Wang et al., 2020	√				√		
Hosseini-Motlagh et al., 2019		√			√		
Mondal and Giri, 2021		√		√	√		
Jiang et al., 2021		√			√		
Panda et al., 2015		√			√		
Yao et al., 2021		√			√		
Cheng and Ding, 2021	√					√	
He et al., 2020	√					√	
Li, 2020	√					√	
Present study		√	√	√		√	√

*S, supplier; M, manufacturer; R, retailer.

The rest of the paper is organized as follows: Section 2 describes the problem and puts forward the model assumptions. Sections 3 to 6 discuss the situation of decentralized decision-making, local alliance decision-making without cost sharing, local alliance decision-making with cost sharing, and overall alliance decision-making. Section 7 compares and analyzes the differences in the optimal level of CSR effort for the supplier, the manufacturer, and the retailer, the optimal value of profit for the supply chain members and the supply chain system, and the expectation and variance of CSR goodwill under four situations. Finally, the results obtained in this paper can provide some theoretical guidance for CSR coordination in supply chain.

## Problem Description and Model Assumptions

It is considered that the three-tier supply chain is formed by a single supplier, a single manufacturer, and a single retailer. Stochastic differential game is used to study the CSR coordination of the supply chain. To improve CSR goodwill of the product and increase demand of the market, the supply chain members need to make CSR efforts. Specifically, the supplier fulfills its social responsibility through technological innovation and reduction in energy consumption. The manufacturer fulfills its social responsibility by developing green industry and protecting environment. The retailer fulfills its social responsibility by publicizing green technology and promoting CSR products. For convenience, we give the following Table 2, which helps readers to understand our problem and models.

**TABLE 2:** Summary of notations.

*G*(*t*)	CSR goodwill of the product at time t
σ[*G*(*t*)]	Random interference factors
*z*(*t*)	Standard Wiener process
*S*(*t*)	CSR effort of the supplier at time t
*M*(*t*)	CSR effort of the manufacturer at time t
*R*(*t*)	CSR effort of the retailer at time t
*D*(*t*)	Market demand of the product at time t
*C*_*S*_(*t*)	CSR effort cost of the supplier at time t
*C*_*M*_(*t*)	CSR effort cost of the manufacturer at time t
*C*_*R*_(*t*)	CSR effort cost of the retailer at time t
β(*t*)	CSR cost subsidy rate
λ_*i*_	Effect of the supply chain member’s CSR effort on CSR goodwill
δ	Decline rate of CSR goodwill
α	Market size
θ	Impact of CSR goodwill on market demand
μ_*i*_	Impact of the supply chain member’s CSR effort on market demand
η_*j*_	CSR effort cost coefficients of the supply chain members
ρ	Discount rate
π_*j*_	Marginal profit of the supply chain members
φ	Incremental profit distribution ratio under local alliance decision- making without cost sharing

*i = 1,2,3 represent the supplier, the manufacturer, and the retailer. *j = S,M,R represent the supplier, the manufacturer, and the retailer.

*Assumptions 1*: CSR goodwill of the product is a dynamic changing process. It is positively affected by CSR effort of the supplier, the manufacturer, and the retailer. Taking into account the natural attenuation of CSR goodwill, the classic advertising goodwill model of Nerlove and Arrow (1962) is adopted. The law of CSR goodwill changes over time is:


(1) $$d\,G\,\left(t\right)=\left[\lambda_{1}\,S\,\left(t\right)+\lambda_{2}\,M\,\left(t\right)+\lambda_{3}\,R\,\left(t\right)-\delta \,G\,\left(t\right)\right]\,d\,t+\sigma \,\left[G\,\left(t\right)\right]\,d\,z\,\left(t\right)$$

where initial CSR goodwill *G*(0) = *G*_0_≥0. λ_1_ > 0, λ_2_ > 0, and λ_3_ > 0 represent the supplier’s, the manufacturer’s, and the retailer’s CSR effort, which has positive impact on CSR goodwill, respectively. δ > 0 represents the decline rate of CSR goodwill, which is usually caused by new product launches and consumer forgetting.

*Assumptions 2*: Referring to the dynamic model of system recycling evolution in Ma and Hu (2018), it is assumed that the random interference factor is proportional to the square root of CSR goodwill. That is $\sigma \,\left[G\,\left(t\right)\right]\,d\,z\,\left(t\right)=\sigma \,\sqrt{G\,\left(t\right)}\,d\,z\,\left(t\right)$.

*Assumptions 3*: Since market demand is positively related to CSR goodwill and CSR effort of the supply chain members, the market demand can be expressed as:


(2) $$D\,\left(t\right)=\alpha +\theta \,G\,\left(t\right)+\mu_{1}\,S\,\left(t\right)+\mu_{2}\,M\,\left(t\right)+\mu_{3}\,R\,\left(t\right)$$

whereθ > 0 reflects that CSR goodwill has positive impact on market demand. μ_1_ > 0, μ_2_ > 0, and μ_3_ > 0 represent that the supplier’s, the manufacturer’s, and the retailer’s CSR effort, which has positive impact on market demand, respectively.

*Assumptions 4*: Referring to the assumption of CSR behavior cost in Ni et al. (2010), it is assumed that CSR effort cost is a convex function of the CSR effort level. So, the CSR effort cost of the supplier, the manufacturer, and the retailer is, respectively,


(3) $$C_{S}\,\left(t\right)=\frac{1}{2}\,\eta_{S}\,S^{2}\,\left(t\right)$$

(4) $$C_{M}\,\left(t\right)=\frac{1}{2}\,\eta_{M}\,M^{2}\,\left(t\right)$$

(5) $$C_{R}\,\left(t\right)=\frac{1}{2}\,\eta_{R}\,R^{2}\,\left(t\right)$$

where η_*S*_ > 0, η_*M*_ > 0, and η_*R*_ > 0, respectively, indicate the CSR effort cost coefficients of the supplier, the manufacturer, and the retailer.

*Assumptions 5*: The supplier, the manufacturer, and the retailer all make rational decisions based on complete information. Inventory cost and out-of-stock cost are not considered.

*Assumptions 6*: The supplier, the manufacturer, and the retailer have the same discount rate ρ > 0 and seek to maximize their own profit in an infinite time frame.

*Assumptions 7*: The marginal profit of the supplier, the manufacturer, and the retailer is positive, respectively, which is π_*S*_ > 0,π_*M*_ > 0,*and*π_*R*_ > 0. In addition, the optimal level of CSR effort for the supplier, the manufacturer, and the retailer is determined by the static feedback control strategy. For the convenience of writing, the time *t* will be omitted in the following text.

## Decentralized Decision-Making

Under decentralized decision-making, the supplier, the manufacturer, and the retailer determine their respective CSR effort simultaneously and maximize their own profit independently. This situation is Nash noncooperative game and is denoted by *N*. At this time, decision-making problems of the supplier, the manufacturer, and the retailer are:


(6) $$\max_{S}J_{S}^{N}=\int_{0}^{\infty }e^{-\rho \,t}\,\left[\pi_{S}\,\left(\alpha +\theta \,G+\mu_{1}\,S+\mu_{2}\,M+\mu_{3}\,R\right)-\frac{1}{2}\,\eta_{S}\,S^{2}\right]\,dt$$

(7) $$\max_{M}J_{M}^{N}=\int_{0}^{\infty }e^{-\rho \,t}\,\left[\pi_{M}\,\left(\alpha +\theta \,G+\mu_{1}\,S+\mu_{2}\,M+\mu_{3}\,R\right)-\frac{1}{2}\,\eta_{M}\,M^{2}\right]\,dt$$

(8) $$\max_{R}J_{R}^{N}=\int_{0}^{\infty }e^{-\rho \,t}\,\left[\pi_{R}\,\left(\alpha +\theta \,G+\mu_{1}\,S+\mu_{2}\,M+\mu_{3}\,R\right)-\frac{1}{2}\,\eta_{R}\,R^{2}\right]\,dt$$

*Proposition 1*: In decentralized decision-making, equilibrium results of the stochastic differential game in the three-tier supply chain are as follows:

(1) The optimal level of CSR effort for the supplier, the manufacturer, and the retailer is:


(9) $$S^{N^{*}}=\frac{\pi_{S}}{\eta_{S}}\,\left(\mu_{1}+\frac{\theta \,\lambda_{1}}{\rho +\delta }\right)$$

(10) $$M^{N^{*}}=\frac{\pi_{M}}{\eta_{M}}\,\left(\mu_{2}+\frac{\theta \,\lambda_{2}}{\rho +\delta }\right)$$

(11) $$R^{N^{*}}=\frac{\pi_{R}}{\eta_{R}}\,\left(\mu_{3}+\frac{\theta \,\lambda_{3}}{\rho +\delta }\right)$$

(2) The optimal value of profit for the supply chain members and the supply chain system is:


(12) VSN*=πS⁢θρ+δ⁢G+πS⁢αρ+πS22⁢ρ⁢ηS⁢(μ1+θ⁢λ1ρ+δ)2+πS⁢πMρ⁢ηM⁢(μ2+θ⁢λ2ρ+δ)2+πS⁢πRρ⁢ηR⁢(μ3+θ⁢λ3ρ+δ)2

(13) VMN*=πM⁢θρ+δ⁢G+πM⁢αρ+πM⁢πSρ⁢ηS⁢(μ1+θ⁢λ1ρ+δ)2+πM22⁢ρ⁢ηM⁢(μ2+θ⁢λ2ρ+δ)2+πM⁢πRρ⁢ηR⁢(μ3+θ⁢λ3ρ+δ)2

(14) VRN*=πR⁢θρ+δ⁢G+πR⁢αρ+πR⁢πSρ⁢ηS⁢(μ1+θ⁢λ1ρ+δ)2+πM⁢πRρ⁢ηM⁢(μ2+θ⁢λ2ρ+δ)2+πR22⁢ρ⁢ηR⁢(μ3+θ⁢λ3ρ+δ)2

(15) $$V_{S\,C}^{N^{*}}=\frac{\left(\pi_{S}+\pi_{M}+\pi_{R}\right)\,\theta }{\rho +\delta }\,G+\frac{\left(\pi_{S}+\pi_{M}+\pi_{R}\right)\,\alpha }{\rho }+\frac{\pi_{S}\,\left(\pi_{S}+2\,\pi_{M}+2\,\pi_{R}\right)}{2\,\rho \,\eta_{S}}\,{\left(\mu_{1}+\frac{\theta \,\lambda_{1}}{\rho +\delta }\right)}^{2}+\frac{\pi_{M}\,\left(2\,\pi_{S}+\pi_{M}+2\,\pi_{R}\right)}{2\,\rho \,\eta_{M}}\,{\left(\mu_{2}+\frac{\theta \,\lambda_{2}}{\rho +\delta }\right)}^{2}+\frac{\pi_{R}\,\left(2\,\pi_{S}+2\,\pi_{M}+\pi_{R}\right)}{2\,\rho \,\eta_{R}}\,{\left(\mu_{3}+\frac{\theta \,\lambda_{3}}{\rho +\delta }\right)}^{2}$$

*Proof*: After the time *t*, the optimal value function of long-term profit for the supplier is $J_{S}^{N^{*}}\,\left(S\right)=e^{-\rho \,t}\,V_{S}^{N^{*}}\,\left(G\right)$. According to the optimal control theory, for any $G>0,V_{S}^{N}\,\left(G\right)$ satisfies Hamilton–Jacobi–Bellman (HJB) equation, that is:


(16) $$\rho V_{S}^{N}\left(G\right)=\max_{S}[\pi_{S}\left(\alpha +\theta G+\mu_{1}S+\mu_{2}M+\mu_{3}R\right)-\frac{1}{2}\,\eta_{S}\,S^{2}+V_{S}^{N^{\prime }}\,\left(G\right)\,\left(\lambda_{1}\,S+\lambda_{2}\,M+\lambda_{3}\,R-\delta \,G\right)+\frac{\sigma^{2}\,\left(G\right)}{2}V_{S}^{N^{\prime \prime }}\left(G\right)]$$

where $V_{S}^{N^{\prime }}\,\left(G\right)$ and $V_{S}^{N^{\prime \prime }}\,\left(G\right)$ represent the first and the second partial derivatives of $V_{S}^{N}\,\left(G\right)$ with respect to *G*. Taking the first-order partial derivative of the function on the right-hand side of (16) with respect to *S* and setting the partial derivative equal to zero, we get the supplier’s CSR effort:


(17) $$S=\frac{\pi_{S}\,\mu_{1}+V_{S}^{N^{\prime }}\,\lambda_{1}}{\eta_{S}}$$

Similarly, after the time *t*, the optimal value function of long-term profit for the manufacturer is $J_{M}^{N^{*}}\,\left(M\right)=e^{-\rho \,t}\,V_{M}^{N^{*}}\,\left(G\right)$. For any $G\geq 0,V_{M}^{N}\,\left(G\right)$ satisfies HJB equation, that is:


(18) $$\rho V_{M}^{N}\left(G\right)=\underset{M}{m\,a\,x}[\pi_{M}\left(\alpha +\theta G+\mu_{1}S+\mu_{2}M+\mu_{3}R\right)-\frac{1}{2}\eta_{M}M^{2}+V_{M}^{N^{\prime }}\left(G\right)\left(\lambda_{1}S+\lambda_{2}M+\lambda_{3}R-\delta G\right)+\frac{\sigma^{2}\,\left(G\right)}{2}V_{M}^{N^{\prime \prime }}\left(G\right)]$$

where $V_{M}^{N^{\prime }}\,\left(G\right)$ and $V_{M}^{N^{\prime \prime }}\,\left(G\right)$ represent the first and the second partial derivatives of $V_{M}^{N}\,\left(G\right)$ with respect to *G*. Taking the first-order partial derivative of the function on the right-hand side of (18) with respect to *M* and setting the partial derivative equal to zero, we get the manufacturer’s CSR effort:


(19) $$M=\frac{\pi_{M}\,\mu_{2}+V_{M}^{N^{\prime }}\,\lambda_{2}}{\eta_{M}}$$

Similarly, after the time *t*, the optimal value function of long-term profit for the retailer is $J_{R}^{N^{*}}\,\left(R\right)=e^{-\rho \,t}\,V_{R}^{N^{*}}\,\left(G\right)$. For any $G\geq 0,V_{R}^{N}\,\left(G\right)$ satisfies HJB equation, that is:


(20) $$\rho V_{R}^{N}\left(G\right)=\underset{R}{m\,a\,x}[\pi_{R}\left(\alpha +\theta G+\mu_{1}S+\mu_{2}M+\mu_{3}R\right)-\frac{1}{2}\eta_{R}R^{2}+V_{R}^{N^{\prime }}\left(G\right)\left(\lambda_{1}S+\lambda_{2}M+\lambda_{3}R-\delta G\right)+\frac{\sigma^{2}\,\left(G\right)}{2}V_{R}^{N^{\prime \prime }}\left(G\right)]$$

where $V_{R}^{N^{\prime }}\,\left(G\right)$ and $V_{R}^{N^{\prime \prime }}\,\left(G\right)$ represent the first and the second partial derivatives of $V_{R}^{N}\,\left(G\right)$ with respect to *G*. Taking the first-order partial derivative of the function on the right-hand side of (20) with respect to *R* and setting the partial derivative equal to zero, we get the retailer’s CSR effort:


(21) $$R=\frac{\pi_{R}\,\mu_{3}+V_{R}^{N^{\prime }}\,\lambda_{3}}{\eta_{R}}$$

Substituting (17), (19), and (21) into (16), we obtain:


$$\rho \,V_{S}^{N}\,\left(G\right)=\left[\pi_{S}\,\theta -V_{S}^{N^{\prime }}\,\left(G\right)\,\delta \right]\,G+\pi_{S}\,\alpha +\frac{{\left[\pi_{S}\,\mu_{1}+V_{S}^{N^{\prime }}\,\left(G\right)\,\lambda_{1}\right]}^{2}}{2\,\eta_{S}}+\frac{\left[\pi_{S}\,\mu_{2}+V_{S}^{N^{\prime }}\,\left(G\right)\,\lambda_{2}\right]\,\left[\pi_{M}\,\mu_{2}+V_{M}^{N^{\prime }}\,\left(G\right)\,\lambda_{2}\right]}{\eta_{M}}$$

(22) $$+\frac{\left[\pi_{S}\,\mu_{3}+V_{S}^{N^{\prime }}\,\left(G\right)\,\lambda_{3}\right]\,\left[\pi_{R}\,\mu_{3}+V_{R}^{N^{\prime }}\,\left(G\right)\,\lambda_{3}\right]}{\eta_{R}}+\frac{\sigma^{2}\,\left(G\right)}{2}\,V_{S}^{N^{\prime \prime }}\,\left(G\right)\;$$

Substituting (17), (19), and (21) into (18), we obtain:


$$\rho \,V_{M}^{N}\,\left(G\right)=\left[\pi_{M}\,\theta -V_{M}^{N^{\prime }}\,\left(G\right)\,\delta \right]\,G+\pi_{M}\,\alpha +\frac{\left[\pi_{M}\,\mu_{1}+V_{M}^{N^{\prime }}\,\left(G\right)\,\lambda_{1}\right]\,\left[\pi_{S}\,\mu_{1}+V_{S}^{N^{\prime }}\,\left(G\right)\,\lambda_{1}\right]}{\eta_{S}}+\frac{{\left[\pi_{M}\,\mu_{2}+V_{M}^{N^{\prime }}\,\left(G\right)\,\lambda_{2}\right]}^{2}}{2\,\eta_{M}}$$

(23) $$+\frac{\left[\pi_{M}\,\mu_{3}+V_{M}^{N^{\prime }}\,\left(G\right)\,\lambda_{3}\right]\,\left[\pi_{R}\,\mu_{3}+V_{R}^{N^{\prime }}\,\left(G\right)\,\lambda_{3}\right]}{\eta_{R}}+\frac{\sigma^{2}\,\left(G\right)}{2}\,V_{M}^{N^{\prime \prime }}\,\left(G\right)$$

Substituting (17), (19), and (21) into (20), we obtain:


$$\rho \,V_{R}^{N}\,\left(G\right)=\left[\pi_{R}\,\theta -V_{R}^{N^{\prime }}\,\left(G\right)\,\delta \right]\,G+\pi_{R}\,\alpha +\frac{\left[\pi_{R}\,\mu_{1}+V_{R}^{N^{\prime }}\,\left(G\right)\,\lambda_{1}\right]\,\left[\pi_{S}\,\mu_{1}+V_{S}^{N^{\prime }}\,\left(G\right)\,\lambda_{1}\right]}{\eta_{S}}+\frac{\left[\pi_{R}\,\mu_{2}+V_{R}^{N^{\prime }}\,\left(G\right)\,\lambda_{2}\right]\,\left[\pi_{M}\,\mu_{2}+V_{M}^{N^{\prime }}\,\left(G\right)\,\lambda_{2}\right]}{\eta_{M}}+$$

(24) $$\frac{{\left[\pi_{R}\,\mu_{3}+V_{R}^{N^{\prime }}\,\left(G\right)\,\lambda_{3}\right]}^{2}}{2\,\eta_{R}}+\frac{\sigma^{2}\,\left(G\right)}{2}\,V_{R}^{N^{\prime \prime }}\,\left(G\right)$$

According to structures of (22), (23), and (24), it is assumed that the linear analytical expressions of the optimal value functions $V_{S}^{N}\,\left(G\right),V_{M}^{N}\,\left(G\right)$ and $V_{R}^{N}\,\left(G\right)$ with respect to *G* are $V_{S}^{N}\,\left(G\right)=a_{1}\,G+a_{2}$, $V_{M}^{N}\,\left(G\right)=b_{1}\,G+b_{2}$, $V_{R}^{N}\,\left(G\right)=c_{1}\,G+c_{2}$, where *a*_1_,*a*_2_,*b*_1_,*b*_2_,*c*_1_,*c*_2_ are parameters to be determined. Substituting $V_{S}^{N}\,\left(G\right),V_{M}^{N}\,\left(G\right)$, $V_{R}^{N}\,\left(G\right)$and their first-order partial derivatives with respect to *G* into (22), (23), (24), we can get the following results through the method of undetermined coefficients.


(25) $$a_{1}=\frac{\pi_{S}\,\theta }{\rho +\delta },b_{1}=\frac{\pi_{M}\,\theta }{\rho +\delta },c_{1}=\frac{\pi_{R}\,\theta }{\rho +\delta }$$

(26) a2=πS⁢αρ+πS22⁢ρ⁢ηS⁢(μ1+θ⁢λ1ρ+δ)2+πS⁢πMρ⁢ηM⁢(μ2+θ⁢λ2ρ+δ)2+πS⁢πRρ⁢ηR⁢(μ3+θ⁢λ3ρ+δ)2   

(27) b2=πM⁢αρ+πM⁢πSρ⁢ηS⁢(μ1+θ⁢λ1ρ+δ)2+πM22⁢ρ⁢ηM⁢(μ2+θ⁢λ2ρ+δ)2+πM⁢πRρ⁢ηR⁢(μ3+θ⁢λ3ρ+δ)2

(28) c2=πR⁢αρ+πR⁢πSρ⁢ηS⁢(μ1+θ⁢λ1ρ+δ)2+πM⁢πRρ⁢ηM⁢(μ2+θ⁢λ2ρ+δ)2+πR22⁢ρ⁢ηR⁢(μ3+θ⁢λ3ρ+δ)2 

Substituting *a_1_*, *b*_*1*,_ and *c_1_* into (17), (19) and (21), we obtain the optimal level of CSR effort for the supplier, the manufacturer, and the retailer as shown in (9), (10), and (11). Substituting *a*_1_,*a*_2_,*b*_1_,*b*_2_,*c*_1_,*c*_2_ into $V_{S}^{N}\,\left(G\right),V_{M}^{N}\,\left(G\right)$, and $V_{R}^{N}\,\left(G\right)$, we obtain the optimal value of profit for the supply chain members and the supply chain system as shown in (12), (13), (14), and (15).

From Proposition 1, we know that the optimal value of profit for the supplier, the manufacturer, and the retailer is related to CSR goodwill. CSR goodwill is affected by various random interference factors, so it is necessary to study the expectation and variance of CSR goodwill.

*Proposition 2*: Under decentralized decision-making, the expectation of CSR goodwill and its stable value are:


(29) $$E\,\left[G_{1}\,\left(t\right)\right]=\frac{\tau_{1}}{\delta }+e^{-\delta \,t}\,\left(G_{0}-\frac{\tau_{1}}{\delta }\right),\underset{t\to \infty }{l\,i\,m}E\,\left[G_{1}\,\left(t\right)\right]=\frac{\tau_{1}}{\delta }$$

the variance of CSR goodwill and its stable value are:


(30) $$D\,\left[G_{1}\,\left(t\right)\right]=\frac{\sigma^{2}\,\left[\tau_{1}-2\,\left(\tau_{1}-\delta \,G_{0}\right)\,e^{-\delta \,t}+\left(\tau_{1}-2\,\delta \,G_{0}\right)\,e^{-2\,\delta \,t}\right]}{2\,\delta^{2}},\underset{t\to \infty }{l\,i\,m}D\,\left[G_{1}\,\left(t\right)\right]=\frac{\sigma^{2}\,\tau_{1}}{2\,\delta^{2}}$$

where $\tau_{1}=\frac{\lambda_{1}\,\pi_{S}}{\eta_{S}}\,\left(\mu_{1}+\frac{\theta \,\lambda_{1}}{\rho +\delta }\right)+\frac{\lambda_{2}\,\pi_{M}}{\eta_{M}}\,\left(\mu_{2}+\frac{\theta \,\lambda_{2}}{\rho +\delta }\right)+\frac{\lambda_{3}\,\pi_{R}}{\eta_{R}}\,\left(\mu_{3}+\frac{\theta \,\lambda_{3}}{\rho +\delta }\right)$.

*Proof*: Substituting (9), (10) and (11) into (1), we obtain:


(31) $$d\,G\,\left(t\right)=\left[\tau_{1}-\delta \,G\,\left(t\right)\right]\,d\,t+\sigma \,\left[G\,\left(t\right)\right]\,d\,z\,\left(t\right)$$

where $\tau_{1}=\frac{\lambda_{1}\,\pi_{S}}{\eta_{S}}\,\left(\mu_{1}+\frac{\theta \,\lambda_{1}}{\rho +\delta }\right)+\frac{\lambda_{2}\,\pi_{M}}{\eta_{M}}\,\left(\mu_{2}+\frac{\theta \,\lambda_{2}}{\rho +\delta }\right)+\frac{\lambda_{3}\,\pi_{R}}{\eta_{R}}\,\left(\mu_{3}+\frac{\theta \,\lambda_{3}}{\rho +\delta }\right)$. Taking the expectation from both sides of (31), we get:


(32) $$d\,E\,\left[G\,\left(t\right)\right]=\left[\tau_{1}-\delta \,E\,\left(G\right)\right]\,d\,t$$

Since *E*[*G*(0)] = *G*_0_, we have:


(33) $$E\,\left[G\,\left(t\right)\right]=\frac{\tau_{1}}{\delta }+e^{-\delta \,t}\,\left(G_{0}-\frac{\tau_{1}}{\delta }\right)$$

That is the expectation of CSR goodwill and its stable value are shown in (29).

By Assumptions 2, we know that $\sigma \,\left[G\,\left(t\right)\right]\,d\,z\,\left(t\right)=\sigma \,\sqrt{G\,\left(t\right)}\,d\,z\,\left(t\right)$. Applying the $I\,t\,\hat{o}$ formula to *G*^2^(*t*), we obtain:


(34) $$d\,G^{2}\,\left(t\right)=\left[\left(2\,\tau_{1}+\sigma^{2}\right)\,G-2\,\delta \,G^{2}\right]\,d\,t+2\,G\,\sigma \,\sqrt{G}\,d\,z\,\left(t\right)$$

Taking the expectation from both sides, we get:


(35) $$d\,E\,\left[G^{2}\,\left(t\right)\right]=\left[\left(2\,\tau_{1}+\sigma^{2}\right)\,E\,\left(G\right)-2\,\delta \,E\,\left(G^{2}\right)\right]\,d\,t$$

Since E⁢[G2⁢(0)]=G02, we have:


(36) E⁢[G2⁢(t)]=(2⁢τ1+σ2)⁢[τ1-2⁢(τ1-δ⁢G0)⁢e-δ⁢t+(τ1-2⁢δ⁢G0)⁢e-2⁢δ⁢t]2⁢δ2+G02⁢e-2⁢δ⁢t

It follows that:


D⁢[G⁢(t)]=E⁢[G2⁢(t)]-{E⁢[G⁢(t)]}2={(2⁢τ1+σ2)⁢[τ1-2⁢(τ1-δ⁢G0)⁢e-δ⁢t+(τ1-2⁢δ⁢G0)⁢e-2⁢δ⁢t]2⁢δ2+G02e-2⁢δ⁢t}-{τ1δ+e-δ⁢t(G0-τ1δ)}2

(37) $$=\frac{\sigma^{2}\,\left[\tau_{1}-2\,\left(\tau_{1}-\delta \,G_{0}\right)\,e^{-\delta \,t}+\left(\tau_{1}-2\,\delta \,G_{0}\right)\,e^{-2\,\delta \,t}\right]}{2\,\delta^{2}}$$

That is the variance of CSR goodwill and its stable value are shown in (30).

**FIGURE 1:**
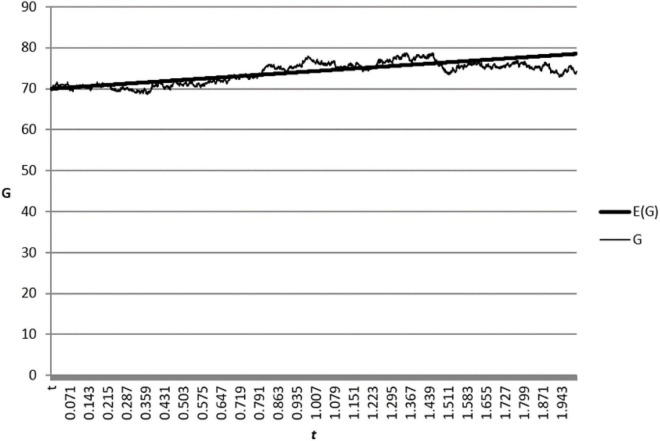
CSR goodwill and its expectation change over time.

Next, we use numerical simulation to illustrate the relationship between CSR goodwill and its expectation. Referring to the simulation method of Prasad and Sethi (2004) and discretizing the formula (31), we can get:


(38) $$G\,\left(t+△\,t\right)=G\,\left(t\right)+\left[\tau_{1}-\delta \,G\,\left(t\right)\right]\,△\,t+\sigma \,\sqrt{G\,\left(t\right)}\,\sqrt{△\,t}\,\zeta \,\left(t\right)\;$$

where ζ(*t*) is a standard normal distribution variable with independent and identical distribution and the step length △*t* = 0.001. Referring to the parameter selection of Zhu et al. (2017), we set benchmark parameters as: *G*_0_ = 70, τ_1_ = 5, δ = 0.01, σ = 0.8, *t*→[0,2]. Substituting the benchmark parameters into (38), we obtain a changed graph of CSR goodwill and its expectation over time, which is shown in Figure 1.

Figure 1 shows that CSR goodwill is a random variable, which always fluctuates up and down its expectations. In fact, it is very difficult for the supply chain members to obtain the exact value of CSR goodwill. The approximate value of CSR goodwill is used in most cases. For this reason, the confidence interval can be used to describe the scope of real CSR goodwill. With a confidence level of 95%, the confidence interval of CSR goodwill is:


(39) $$\left\{E\,\left[G\,\left(t\right)\right]-1.96\,\sqrt{D\,\left[G\,\left(t\right)\right]},E\,\left[G\,\left(t\right)\right]+1.96\,\sqrt{D\,\left[G\,\left(t\right)\right]}\right\}$$

At any time, the supply chain members can use (39) to calculate the scope of CSR goodwill with confidence level of 95%, which can help the supply chain members to make CSR decisions.

## Local Alliance Decision-Making Without Cost Sharing

If a binding profit distribution agreement is reached in advance, the supply chain members are likely to form local alliance. It is assumed that the enterprise only alliances with neighboring enterprises, such as the alliance of the supplier and the manufacturer or the alliance of the manufacturer and the retailer. Because there is no difference between these two alliances (Zhang et al., 2015), so we only study the alliance composed of the supplier and the manufacturer. At this time, aiming at maximizing the profit of the local alliance, the supplier and the manufacturer negotiate their respective CSR effort level. The retailer determines its CSR effort independently to maximize its own profit. This situation is local alliance decision-making without cost sharing and is denoted by *S_1_*. Decision-making problems of the local alliance and the retailer are:


$$\max_{S,M}J_{S\,M}^{S_{1}}=\int_{0}^{\infty }e^{-\rho \,t}[\left(\pi_{S}+\pi_{M}\right)\left(\alpha +\theta G+\mu_{1}S+\mu_{2}M+\mu_{3}R\right)-\frac{1}{2}\eta_{S}S^{2}-\frac{1}{2}\eta_{M}M^{2}]dt$$

$$\max_{R}J_{R}^{S_{1}}=\int_{0}^{\infty }e^{-\rho \,t}[\pi_{R}\left(\alpha +\theta G+\mu_{1}S+\mu_{2}M+\mu_{3}R\right)-\frac{1}{2}\eta_{R}R^{2}]dt$$

*Proposition 3*: Under local alliance decision-making without cost sharing, equilibrium results of the stochastic differential game in three-tier supply chain are as follows:

(1) The optimal level of CSR effort for the supplier, the manufacturer, and the retailer is:


SS1*=πS+πMηS⁢(μ1+θ⁢λ1ρ+δ)

MS1*=πS+πMηM⁢(μ2+θ⁢λ2ρ+δ)

RS1*=πRηR⁢(μ3+θ⁢λ3ρ+δ)

(2) The optimal value of profit for the local alliance, the retailer, and the supply chain system is:


VS⁢MS1*=(πS+πM)⁢θρ+δ⁢G+(πS+πM)⁢αρ+(πS+πM)22⁢ρ⁢ηS⁢(μ1+θ⁢λ1ρ+δ)2+(πS+πM)22⁢ρ⁢ηM⁢(μ2+θ⁢λ2ρ+δ)2+πR⁢(πS+πM)ρ⁢ηR⁢(μ3+θ⁢λ3ρ+δ)2 

VRS1*=πR⁢θρ+δ⁢G+πR⁢αρ+πR⁢(πS+πM)ρ⁢ηS⁢(μ1+θ⁢λ1ρ+δ)2+πR⁢(πS+πM)ρ⁢ηM⁢(μ2+θ⁢λ2ρ+δ)2+πR22⁢ρ⁢ηR⁢(μ3+θ⁢λ3ρ+δ)2  

VS⁢CS1*=(πS+πM+πR)⁢θρ+δ⁢G+(πS+πM+πR)⁢αρ+πS2+πM2+2⁢πS⁢πM+2⁢πS⁢πR+2⁢πM⁢πR2⁢ρ⁢ηS⁢(μ1+θ⁢λ1ρ+δ)2+πS2+πM2+2⁢πS⁢πM+2⁢πS⁢πR+2⁢πM⁢πR2⁢ρ⁢ηM⁢(μ2+θ⁢λ2ρ+δ)2

+πR2+2⁢πS⁢πR+2⁢πM⁢πR2⁢ρ⁢ηR⁢(μ3+θ⁢λ3ρ+δ)2

The proof of Proposition 3 is similar to that of Proposition 1, so it is omitted here. From Proposition 3, we know that the optimal value of profit for the local alliance and the retailer is related to CSR goodwill. CSR goodwill is affected by various random interference factors, so it is necessary for us to study the expectation and variance of CSR goodwill.

*Proposition 4*: Under local alliance decision-making without cost sharing, the expectation of CSR goodwill and its stable value are:


$$E\,\left[G_{2}\,\left(t\right)\right]=\frac{\tau_{2}}{\delta }+e^{-\delta \,t}\,\left(G_{0}-\frac{\tau_{2}}{\delta }\right),\underset{t\to \infty }{l\,i\,m}E\,\left[G_{2}\,\left(t\right)\right]=\frac{\tau_{2}}{\delta }\;$$

the variance of CSR goodwill and its stable value are:


$$D\,\left[G_{2}\,\left(t\right)\right]=\frac{\sigma^{2}\,\left[\tau_{2}-2\,\left(\tau_{2}-\delta \,G_{0}\right)\,e^{-\delta \,t}+\left(\tau_{2}-2\,\delta \,G_{0}\right)\,e^{-2\,\delta \,t}\right]}{2\,\delta^{2}},\underset{t\to \infty }{l\,i\,m}D\,\left[G_{2}\,\left(t\right)\right]=\frac{\sigma^{2}\,\tau_{2}}{2\,\delta^{2}}$$

where, $\tau_{2}=\frac{\lambda_{1}\,\left(\pi_{S}+\pi_{M}\right)}{\eta_{S}}\,\left(\mu_{1}+\frac{\theta \,\lambda_{1}}{\rho +\delta }\right)+\frac{\lambda_{2}\,\left(\pi_{S}+\pi_{M}\right)}{\eta_{M}}\,\left(\mu_{2}+\frac{\theta \,\lambda_{2}}{\rho +\delta }\right)+\frac{\lambda_{3}\,\pi_{R}}{\eta_{R}}\,\left(\mu_{3}+\frac{\theta \,\lambda_{3}}{\rho +\delta }\right)$.

The proof of Proposition 4 is similar to that of Proposition 2, so it is omitted here.

Under local alliance decision-making without cost sharing, according to the incremental profit distribution agreement between the supplier and the manufacturer based on decentralized decision-making, the optimal value of profit for the local alliance is reasonably distributed. Based on the profit obtained by both parties under decentralized decision-making, the incremental profit is divided according to CSR effort. The incremental profit distribution ratio for the supplier is φ and the incremental profit distribution ratio for the manufacturer is 1−φ, where:


$$\varphi =\frac{1}{\eta_{S}}\,\left(\mu_{1}+\frac{\theta \,\lambda_{1}}{\rho +\delta }\right)/\left[\frac{1}{\eta_{S}}\,\left(\mu_{1}+\frac{\theta \,\lambda_{1}}{\rho +\delta }\right)+\frac{1}{\eta_{M}}\,\left(\mu_{2}+\frac{\theta \,\lambda_{2}}{\rho +\delta }\right)\right]$$

Since the incremental profit of the local alliance is:


VS⁢MS1*-VSN*-VMN*=πM22⁢ρ⁢ηS⁢(μ1+θ⁢λ1ρ+δ)2+πS22⁢ρ⁢ηM⁢(μ2+θ⁢λ2ρ+δ)2

we have that the incremental profit of the supplier is:


φ⁢[πM22⁢ρ⁢ηS⁢(μ1+θ⁢λ1ρ+δ)2+πS22⁢ρ⁢ηM⁢(μ2+θ⁢λ2ρ+δ)2]

and the incremental profit of the manufacturer is:


(1-φ)⁢[πM22⁢ρ⁢ηS⁢(μ1+θ⁢λ1ρ+δ)2+πS22⁢ρ⁢ηM⁢(μ2+θ⁢λ2ρ+δ)2]

Furthermore, the optimal value of profit for the supplier is:


VSS1*=πS⁢θρ+δ⁢G+πS⁢αρ+πS2+φ1⁢πM22⁢ρ⁢ηS⁢(μ1+θ⁢λ1ρ+δ)2+2⁢πS⁢πM+φ1⁢πS22⁢ρ⁢ηM⁢(μ2+θ⁢λ2ρ+δ)2+πS⁢πRρ⁢ηR⁢(μ3+θ⁢λ3ρ+δ)2

and the optimal value of profit for the manufacturer is:


VMS1*=πM⁢θρ+δ⁢G+πM⁢αρ+2⁢πM⁢πS+(1-φ1)⁢πM22⁢ρ⁢ηS(μ1+θ⁢λ1ρ+δ)2+πM2+(1-φ1)⁢πS22⁢ρ⁢ηM⁢(μ2+θ⁢λ2ρ+δ)2+πM⁢πRρ⁢ηR⁢(μ3+θ⁢λ3ρ+δ)2

## Local Alliance Decision-Making With Cost Sharing

Under local alliance decision-making with cost sharing, if a binding profit distribution agreement is reached in advance, the supplier and the manufacturer will form a local alliance to occupy a dominant position in the supply chain. To stimulate the retailer to invest more in CSR effort, the local alliance is willing to provide CSR cost subsidy for the retailer. Suppose that CSR cost subsidy rate is denoted by β(*t*), where 0≤β(*t*)≤1. The decision-making process is as follows: the supplier and the manufacturer determine the CSR effort level and the CSR cost subsidy rate for the retailer through negotiation. The retailer determines its own CSR effort level based on the alliance decision of the supplier and the manufacturer. From a long-term dynamic perspective, the local alliance and the retailer form a Stackelberg noncooperative game, which is denoted by *S_2_*. At this time, decision-making problems of the local alliance and the retailer are:


$$\max_{S,M}J_{S\,M}^{S_{2}}=\int_{0}^{\infty }e^{-\rho \,t}[\left(\pi_{S}+\pi_{M}\right)\left(\alpha +\theta G+\mu_{1}S+\mu_{2}M+\mu_{3}R\right)\;-\frac{1}{2}\eta_{S}S^{2}-\frac{1}{2}\eta_{M}M^{2}-\frac{1}{2}\beta \eta_{R}R^{2}]dt$$

$$\max_{R}J_{R}^{S_{2}}=\int_{0}^{\infty }e^{-\rho \,t}[\pi_{R}\left(\alpha +\theta G+\mu_{1}S+\mu_{2}M+\mu_{3}R\right)\;-\frac{1}{2}\left(1-\beta \right)\eta_{R}R^{2}]dt$$

*Proposition 5*: In local alliance decision-making with cost sharing, equilibrium results of the stochastic differential game in three-tier supply chain are as follows:

(1) The optimal level of CSR effort for the supplier, the manufacturer, and the retailer is:


SS2*=πS+πMηS⁢(μ1+θ⁢λ1ρ+δ)

MS2*=πS+πMηM⁢(μ2+θ⁢λ2ρ+δ)

RS2*=2⁢(πS+πM)+πR2⁢ηR⁢(μ3+θ⁢λ3ρ+δ)

the optimal CSR cost subsidy rate is:


$$\beta^{*}\,\left\{ \begin{array}{c} \frac{2\,\left(\pi_{S}+\pi_{M}\right)-\pi_{R}}{2\,\left(\pi_{S}+\pi_{M}\right)+\pi_{R}},\text{when}\,\pi_{S}+\pi_{M}>\frac{1}{2}\,\pi_{R},\\ 0,\mathrm{when}\,\pi_{S}+\pi_{M}\leq \frac{1}{2}\,\pi_{R} \end{array}\right.$$

(2) The optimal value of profit for the local alliance, the retailer, and the supply chain system is:


VS⁢MS2*=(πS+πM)⁢θρ+δ⁢G+(πS+πM)⁢αρ+(πS+πM)22⁢ρ⁢ηS(μ1+θ⁢λ1ρ+δ)2+(πS+πM)22⁢ρ⁢ηM⁢(μ2+θ⁢λ2ρ+δ)2+[2⁢(πS+πM)+πR]28⁢ρ⁢ηR⁢(μ3+θ⁢λ3ρ+δ)2 

VRS2*=πR⁢θρ+δ⁢G+πR⁢αρ+πR⁢(πS+πM)ρ⁢ηS⁢(μ1+θ⁢λ1ρ+δ)2+πR⁢(πS+πM)ρ⁢ηM⁢(μ2+θ⁢λ2ρ+δ)2+πR⁢[2⁢(πS+πM)+πR]4⁢ρ⁢ηR⁢(μ3+θ⁢λ3ρ+δ)2

VS⁢CS2*=(πS+πM+πR)⁢θρ+δ⁢G+(πS+πM+πR)⁢αρ+πS2+πM2+2⁢πS⁢πM+2⁢πS⁢πR+2⁢πM⁢πR2⁢ρ⁢ηS⁢(μ1+θ⁢λ1ρ+δ)2

+πS2+πM2+2⁢πS⁢πM+2⁢πS⁢πR+2⁢πM⁢πR2⁢ρ⁢ηM⁢(μ2+θ⁢λ2ρ+δ)2+[2⁢(πS+πM)+πR]⁢[2⁢(πS+πM)+3⁢πR]8⁢ρ⁢ηR⁢(μ3+θ⁢λ3ρ+δ)2

Proposition 5 can be proved by backward induction. First, we can take first-order partial derivative of the optimal value function of profit for the retailer $V_{R}^{S_{2}}\,\left(G\right)$ with respect to *R*. Second, we calculate first-order partial derivative of the optimal value function of profit for local alliance $V_{S\,M}^{S_{2}}\,\left(G\right)$ with respect to *S*,*M*andβ, respectively. The proof of Proposition 5 is similar to that of Proposition 1, so it is omitted here. From Proposition 5, we know that the optimal value of profit for the local alliance and the retailer is related to CSR goodwill. CSR goodwill is affected by various random interference factors, so it is necessary for us to study the expectation and variance of CSR goodwill.

*Proposition 6*: In local alliance decision-making with cost sharing, the expectation of CSR goodwill and its stable value are:


$$E\,\left[G_{3}\,\left(t\right)\right]=\frac{\tau_{3}}{\delta }+e^{-\delta \,t}\,\left(G_{0}-\frac{\tau_{3}}{\delta }\right),\underset{t\to \infty }{l\,i\,m}E\,\left[G_{3}\,\left(t\right)\right]=\frac{\tau_{3}}{\delta }\;$$

the variance of CSR goodwill and its stable value are:


$$D\,\left[G_{3}\,\left(t\right)\right]=\frac{\sigma^{2}\,\left[\tau_{3}-2\,\left(\tau_{3}-\delta \,G_{0}\right)\,e^{-\delta \,t}+\left(\tau_{3}-2\,\delta \,G_{0}\right)\,e^{-2\,\delta \,t}\right]}{2\,\delta^{2}},\underset{t\to \infty }{l\,i\,m}D\,\left[G_{3}\,\left(t\right)\right]=\frac{\sigma^{2}\,\tau_{3}}{2\,\delta^{2}}$$

where $\tau_{3}=\frac{\lambda_{1}\,\left(\pi_{S}+\pi_{M}\right)}{\eta_{S}}\,\left(\mu_{1}+\frac{\theta \,\lambda_{1}}{\rho +\delta }\right)+\frac{\lambda_{2}\,\left(\pi_{S}+\pi_{M}\right)}{\eta_{M}}\,\left(\mu_{2}+\frac{\theta \,\lambda_{2}}{\rho +\delta }\right)+\frac{\lambda_{3}\,\left[2\,\left(\pi_{S}+\pi_{M}\right)+\pi_{R}\right]}{2\,\eta_{R}}\,\left(\mu_{3}+\frac{\theta \,\lambda_{3}}{\rho +\delta }\right)$.

The proof of Proposition 6 is similar to that of Proposition 2, so it is omitted here.

In local alliance decision-making with cost sharing, profit incremental distribution agreement based on decentralized decision-making reached between supplier and manufacturer in advance. Then, they distribute the optimal value of profit for the local alliance reasonably. Based on the profit obtained by both parties under decentralized decision-making, the incremental profit is divided according to CSR effort. Similar to the profit distribution method in Section 4, the optimal value of profit for the supplier and the manufacturer can be obtained.

## Overall Alliance Decision-Making

If a binding profit distribution agreement is reached in advance, the supply chain members may form an overall alliance. In overall alliance decision-making, the supplier, the manufacturer, and the retailer make joint decisions to maximize the profit of the overall alliance. This situation is cooperative game and is denoted by *C*. At this time, decision-making problem of the overall alliance is:


$$\max_{S,M,R}J_{S\,C}^{C}=\int_{0}^{\infty }e^{-\rho \,t}[\left(\pi_{S}+\pi_{M}+\pi_{R}\right)(\alpha +\theta G\;+\mu_{1}S+\mu_{2}M+\mu_{3}R)-\frac{1}{2}\eta {}_{S}S{}^{2}-\frac{1}{2}\eta {}_{M}M{}^{2}\;-\frac{1}{2}\eta_{R}R^{2}]dt$$

*Proposition 7*: In overall alliance decision-making, equilibrium results of the stochastic differential game in three-tier supply chain are as follows:

1. The optimal level of CSR effort for the supplier, the manufacturer, and the retailer is:

$$S^{C^{*}}=\frac{\pi_{S}+\pi_{M+}\,\pi_{R}}{\eta_{S}}\,\left(\mu_{1}+\frac{\theta \,\lambda_{1}}{\rho +\delta }\right)$$

$$M^{C^{*}}=\frac{\pi_{S}+\pi_{M+}\,\pi_{R}}{\eta_{M}}\,\left(\mu_{2}+\frac{\theta \,\lambda_{2}}{\rho +\delta }\right)$$

$$R^{C^{*}}=\frac{\pi_{S}+\pi_{M+}\,\pi_{R}}{\eta_{R}}\,\left(\mu_{3}+\frac{\theta \,\lambda_{3}}{\rho +\delta }\right)$$

(2) The optimal value of profit for the overall alliance is:


$$V_{S\,C}^{C^{*}}=\frac{\left(\pi_{S}+\pi_{M}+\pi_{R}\right)\,\theta }{\rho +\delta }\,G+\frac{\left(\pi_{S}+\pi_{M}+\pi_{R}\right)\,\alpha }{\rho }+\frac{{\left(\pi_{S}+\pi_{M}+\pi_{R}\right)}^{2}}{2\,\rho \,\eta_{S}}\,{\left(\mu_{1}+\frac{\theta \,\lambda_{1}}{\rho +\delta }\right)}^{2}+\frac{{\left(\pi_{S}+\pi_{M}+\pi_{R}\right)}^{2}}{2\,\rho \,\eta_{M}}\,{\left(\mu_{2}+\frac{\theta \,\lambda_{2}}{\rho +\delta }\right)}^{2}+\frac{{\left(\pi_{S}+\pi_{M}+\pi_{R}\right)}^{2}}{2\,\rho \,\eta_{R}}\,{\left(\mu_{3}+\frac{\theta \,\lambda_{3}}{\rho +\delta }\right)}^{2}\;$$

The proof of Proposition 7 is similar to that of Proposition 1, so it is omitted here. From Proposition 7, we know that the optimal value of profit for the overall alliance is related to CSR goodwill. Since CSR goodwill is affected by various random interference factors, it is necessary for us to study the expectation and variance of CSR goodwill.

*Proposition 8*: In overall alliance decision-making, the expectation of CSR goodwill and its stable value are:


$$E\,\left[G_{4}\,\left(t\right)\right]=\frac{\tau_{4}}{\delta }+e^{-\delta \,t}\,\left(G_{0}-\frac{\tau_{4}}{\delta }\right),\underset{t\to \infty }{l\,i\,m}E\,\left[G_{4}\,\left(t\right)\right]=\frac{\tau_{4}}{\delta }$$

the variance of CSR goodwill and its stable value are:


$$D\,\left[G_{4}\,\left(t\right)\right]=\frac{\sigma^{2}\,\left[\tau_{4}-2\,\left(\tau_{4}-\delta \,G_{0}\right)\,e^{-\delta \,t}+\left(\tau_{4}-2\,\delta \,G_{0}\right)\,e^{-2\,\delta \,t}\right]}{2\,\delta^{2}},\underset{t\to \infty }{l\,i\,m}D\,\left[G_{4}\,\left(t\right)\right]=\frac{\sigma^{2}\,\tau_{4}}{2\,\delta^{2}}$$

where, $\tau_{4}=\frac{\lambda_{1}\,\left(\pi_{S}+\pi_{M}+\pi_{R}\right)}{\eta_{S}}\,\left(\mu_{1}+\frac{\theta \,\lambda_{1}}{\rho +\delta }\right)+\frac{\lambda_{2}\,\left(\pi_{S}+\pi_{M}+\pi_{R}\right)}{\eta_{M}}$

$\left(\mu_{2}+\frac{\theta \,\lambda_{2}}{\rho +\delta }\right)+\frac{\lambda_{3}\,\left(\pi_{S}+\pi_{M}+\pi_{R}\right)}{\eta_{R}}\,\left(\mu_{3}+\frac{\theta \,\lambda_{3}}{\rho +\delta }\right)$.

The proof of Proposition 8 is similar to that of Proposition 2, so it is omitted here.

## Comparison and Analysis

Through the analysis from Sections 3 to 6, we obtain the optimal level of CSR effort for the supplier, the manufacturer, and the retailer, the optimal value of profit for the supply chain members and the supply chain system, and the expectation and variance of CSR goodwill under different situations. In this section, we will compare these results and draw some important conclusions.

*Corollary 1*: Compared with decentralized decision-making, under local alliance decision-making without cost sharing:

(1) The optimal level of CSR effort increases for the supplier and the manufacturer, whereas the optimal level of CSR effort remains unchanged for the retailer, that is SN*<SS1*, MN*<MS1*, RN*=RS1*.
(2) The optimal value of profit increases for the retailer and the supply chain system, that is, VRN*<VRS1*, VS⁢CN*<VS⁢CS1*. If the supplier and the manufacturer can reach the profit incremental distribution agreement based on decentralized decision-making in advance, the optimal value of profit increases for the supplier and the manufacturer, that is, VSN*<VSS1*, VMN*<VMS1*.
(3) The expectation of CSR goodwill and its stable value increase, that is, *E*[*G*_1_(*t*)]≤*E*[*G*_2_(*t*)], *lim*_*t*→∞_*E*[*G*_1_(*t*)] < *lim*_*t*→∞_*E*[*G*_2_(*t*)]. The variance of CSR goodwill and its stable value increase, that is, *D*[*G*_1_(*t*)]≤*D*[*G*_2_(*t*)],*lim*_*t*→∞_*D*[*G*_1_(*t*)] < *lim*_*t*→∞_*D*[*G*_2_(*t*)].

*Proof*: It is easy to know from Proposition 1 (1) and Proposition 3 (1) that Corollary 1 (1) is true. From Proposition 1 (2), Proposition 3 (2), and the profit distribution results in Section 4, we have that Corollary 1 (2) is true. From Proposition 2 and Proposition 4, we know that:


$$E\,\left[G_{1}\,\left(t\right)\right]\leq E\,\left[G_{2}\,\left(t\right)\right],\underset{t\to \infty }{l\,i\,m}E\,\left[G_{1}\,\left(t\right)\right]<\underset{t\to \infty }{l\,i\,m}E\,\left[G_{2}\,\left(t\right)\right]$$

Furthermore,


$$D\,\left[G_{2}\,\left(t\right)\right]-D\,\left[G_{1}\,\left(t\right)\right]=\frac{\sigma^{2}\,\left(\tau_{2}-\tau_{1}\right)}{2\,\delta^{2}}\,\left(1-2\,e^{-\delta \,t}+e^{-2\,\delta \,t}\right)=\frac{\sigma^{2}}{2\,\delta^{2}}\,\left[\frac{\lambda_{1}\,\pi_{M}}{\eta_{S}}\,\left(\mu_{1}+\frac{\theta \,\lambda_{1}}{\rho +\delta }\right)+\frac{\lambda_{2}\,\pi_{S}}{\eta_{M}}\,\left(\mu_{2}+\frac{\theta \,\lambda_{2}}{\rho +\delta }\right)\right]\left(1-2\,e^{-\delta \,t}+e^{-2\,\delta \,t}\right)$$

$$\underset{t\to \infty }{l\,i\,m}D\,\left[G_{2}\,\left(t\right)\right]-\underset{t\to \infty }{l\,i\,m}D\,\left[G_{1}\,\left(t\right)\right]=\frac{\sigma^{2}\,\left(\tau_{2}-\tau_{1}\right)}{2\,\delta^{2}}=\frac{\sigma^{2}}{2\,\delta^{2}}\left[\frac{\lambda_{1}\,\pi_{M}}{\eta_{S}}\,\left(\mu_{1}+\frac{\theta \,\lambda_{1}}{\rho +\delta }\right)+\frac{\lambda_{2}\,\pi_{S}}{\eta_{M}}\,\left(\mu_{2}+\frac{\theta \,\lambda_{2}}{\rho +\delta }\right)\right]>0$$

Obviously, *lim*_*t*→∞_*D*[*G*_1_(*t*)] < *limt*_*t*→∞_*D*[*G*_2_(*t*)]. Let *f*(*t*) = 1−2*e*^−δ*t*^ + *e*^−2δ*t*^, and then we have:


$$f^{\prime }\,\left(t\right)=\frac{d\,f\,\left(t\right)}{d\,t}=2\,\delta \,e^{-\delta \,t}\,\left(1-e^{-\delta \,t}\right)$$

Thus, for any *t* ∈ [0,∞),*f*′(*t*)≥0 and *f*(0) = 0. Therefore, for any *t* ∈ [0,∞),*f*(*t*)≥0. It follows that *D*[*G*_1_(*t*)]≤*D*[*G*_2_(*t*)].

Compared with decentralized decision-making, under local alliance decision-making without cost sharing, the supplier and the manufacturer can more effectively divide the profit of the local alliance through negotiation and thus improve the optimal level of CSR effort. The retailer makes decision independently without participating in the alliance, and the retailer’s optimal level of CSR effort is not related to whether the supplier and the manufacturer are in alliance. So, the optimal level of CSR effort for the retailer remains unchanged. Since the improvement in the optimal level of CSR effort for the supplier and the manufacturer has a positive impact on CSR goodwill and market demand, the optimal value of profit for the retailer and the supply chain system are all increase. If the supplier and the manufacturer can reach the profit incremental distribution agreement based on decentralized decision-making in advance, then the optimal value of profit increases for the supplier and the manufacturer, and the coordination of the supply chain can be realized. Since the improvement in the optimal level of CSR effort for the supplier and the manufacturer has a positive impact on CSR goodwill, the expectation of CSR goodwill increases. In addition, due to the influence of random interference factors, such as the industry characteristics of the supplier and the manufacturer, market characteristics, government relations, natural environment, and other factors, the local alliance has great uncertainty, so the variance of CSR goodwill increases. This also shows that high profit is accompanied by high risk. In local alliance decision without cost sharing, the supplier, the manufacturer, and the retailer get higher profit, but take greater risk.

*Corollary 2*: Compared with local alliance decision-making without cost sharing, under local alliance decision-making with cost sharing:

(1) the optimal level of CSR effort remains unchanged for the supplier and the manufacturer, that is, SS1*=SS2*,MS1*=MS2*. When $\pi_{S}+\pi_{M}>\frac{1}{2}\,\pi_{R}$, the optimal level of CSR effort increases for the retailer, that is, RS1*<RS2*.
(2) When $\pi_{S}+\pi_{M}>\frac{1}{2}\,\pi_{R}$, the optimal value of profit increases for the retailer and the supply chain system, that is, VRS1*<VRS2*, VS⁢CS1*<VS⁢CS2*. If the supplier and the manufacturer can reach the profit incremental distribution agreement based on decentralized decision-making in advance, then the optimal value of profit increases for the supplier and the manufacturer, that is, VSS1*<VSS2*, VMS1*<VMS2*.
(3) When $\pi_{S}+\pi_{M}>\frac{1}{2}\,\pi_{R}$, the expectation of CSR goodwill and its stable value increase, that is, *E*[*G*_2_(*t*)]≤*E*[*G*_3_(*t*)], *lim*_*t*→∞_*E*[*G*_2_(*t*)]≤*lim*_*t*→∞_*E*[*G*_3_(*t*)]. The variance of CSR goodwill and its stable value increase, that is, *D*[*G*_2_(*t*)]≤*D*[*G*_3_(*t*)], *lim*_*t*→∞_*D*[*G*_2_(*t*)] < *lim*_*t*→∞_*D*[*G*_3_(*t*)].

The proof of Corollary 2 is similar to that of Corollary 1, so it is omitted here.

Compared with local alliance decision-making without cost sharing, under local alliance decision-making with cost sharing, the alliance composed of the supplier and the manufacturer is the core force in the supply chain. Due to the lack of external incentives, the optimal level of CSR effort for both parties remains unchanged. When $\pi_{S}+\pi_{M}>\frac{1}{2}\,\pi_{R}$, the local alliance shares CSR cost for the retailer, which encourages the retailer to take social responsibility. Therefore, the optimal level of CSR effort increases for the retailer. Since the improvement in the optimal level of CSR effort for the retailer has a positive impact on CSR goodwill and market demand, the optimal value of profit for the retailer and the supply chain system will increase when $\pi_{S}+\pi_{M}>\frac{1}{2}\,\pi_{R}$. If the supplier and the manufacturer can reach the profit incremental distribution agreement based on decentralized decision-making in advance, the optimal value of profit increases for the supplier and the manufacturer, and the supply chain coordination can be realized. Since the improvement in the optimal level of CSR effort for the retailer has a positive impact on CSR goodwill, the expectation of CSR goodwill increases. In addition, due to the influence of random interference factors and marginal profit of the supply chain members, the uncertainty of CSR cost subsidy rate increases, so the variance of CSR goodwill increases. This also shows that high profit is accompanied by high risk. In local alliance decision-making with cost sharing, the supplier, the manufacturer and the retailer get higher profit, but take greater risk.

*Corollary 3*: Compared with local alliance decision-making with cost sharing, under overall alliance decision-making:

(1) The optimal level of CSR effort increases for the supplier, the manufacturer and the retailer, that is., SS2*<SC*, MS2*<MC*, RS2*<RC*.
(2) The optimal value of profit increases for overall alliance, that is, VS⁢CS2*<VS⁢CC*.
(3) The expectation of CSR goodwill and its stable value increase, that is, *E*[*G*_3_(*t*)]≤*E*[*G*_4_(*t*)],*lim*_*t*→∞_*E*[*G*_3_(*t*)] < *lim*_*t*→∞_*E*[*G*_4_(*t*)]. The variance of CSR goodwill and its stable value increase, that is, *D*[*G*_3_(*t*)]≤*D*[*G*_4_(*t*)], *lim*_*t*→∞_*D*[*G*_3_(*t*)] < *lim*_*t*→∞_*D*[*G*_4_(*t*)].

The proof of Corollary 3 is similar to that of Corollary 1, so it is omitted here.

Compared with local alliance decision-making with cost sharing, the supply chain members are as a whole in overall alliance decision-making. CSR effort of three parties is complementary and jointly affects the decision-making of three parties. Therefore, the optimal level of CSR effort increases for the supplier, the manufacturer, and the retailer. Since the improvement in the optimal level of CSR effort for the supplier, the manufacturer, and the retailer has a positive impact on CSR goodwill and market demand, the optimal value of profit for overall alliance will increase. Since the improvement in the optimal level of CSR effort for the supplier, the manufacturer, and the retailer has a positive impact on CSR goodwill, the expectation of CSR goodwill increases. In addition, the overall alliance has greater uncertainty due to the influence of random interference factors, such as the industry characteristics of the supplier, the manufacturer, and the retailer, market characteristics, government relations, natural environment, and other factors, so the variance of CSR goodwill increases. This also shows that high profit is accompanied by high risk. In overall alliance decision-making, the supplier, the manufacturer, and the retailer get higher profit, but take greater risk.

Compared with local alliance decision-making with cost sharing, in overall alliance decision-making, the optimal value of profit for overall alliance increases. However, only when the profit of the supplier, the manufacturer, and the retailer all increases, they will choose to cooperate. So, the profit of three parties must satisfy VSS2*<VSC*, VMS2*<VMC*, VRS2*<VRC*. In this paper, we introduce a profit incremental distribution method based on local alliance decision-making with cost sharing. Specifically, based on the profit obtained by three parties under local alliance decision-making with cost sharing, the incremental profit is divided according to CSR effort. Similar to the profit distribution method in Section 4, the optimal value of profit for the supplier, the manufacturer, and the retailer can be obtained. According to this method, the optimal value of profit increases for the supplier, the manufacturer, and the retailer, and the supply chain coordination can be realized.

## Conclusion

It is considered that the three-tier supply chain is formed by a single supplier, a single manufacturer, and a single retailer. Stochastic differential game is used to study the CSR coordination of the supply chain. Under decentralized decision-making, local alliance decision-making without cost sharing, local alliance decision-making with cost sharing, and overall alliance decision-making, we, respectively, calculate the optimal level of CSR effort for the supplier, the manufacturer, and the retailer, the optimal value of profit for the supply chain members and the supply chain system, and the expectation and variance of CSR goodwill and obtain the following important results:

(1) The changes in relevant indexes under the local alliance decision-making without cost sharing are obtained by comparing the decentralized decision-making with the local alliance decision-making without cost sharing. For the optimal level of CSR effort about the supply chain members, both the supplier and the manufacturer improve, and the retailer remains unchanged. For the supply chain member’s profit and the total system profit, both the retailer and the supply chain system increase. If the supplier and the manufacturer reach the profit incremental distribution agreement based on the decentralized decision-making in advance, the supplier and the manufacturer also improve. For CSR goodwill, both expectation and variance are increased. Therefore, in the process of supply chain CSR management, the supplier and the manufacturer should reach an agreement on profit increment distribution. Furthermore, we can coordinate the supply chain and improve CSR management level of the supply chain.
(2) The changes in relevant indexes under the local alliance decision-making with cost sharing are obtained by comparing the local alliance decision-making without cost sharing with the local alliance decision-making with cost sharing. For the optimal level of CSR effort about the supply chain members, the supplier and manufacturer remain the same. When the sum of the marginal profit of the supplier and the manufacturer is more than half of the retailer’s marginal profit, the retailer’s optimal level of CSR effort is improved. For the supply chain member’s profit and the total system profit, both the retailer and the supply chain system increase. If the supplier and the manufacturer reach the profit incremental distribution agreement based on decentralized decision-making in advance, the profit of the supplier and the manufacturer also increases. For CSR goodwill, the expectation and variance are increased. Therefore, in the process of supply chain CSR management, the supplier and manufacturer should reach an agreement on profit increment distribution and cooperate with CSR management. At the same time, the local alliance should subsidize the retailer’s CSR cost to improve the overall level of the supply chain.
(3) By comparing the local alliance decision-making with cost sharing with the overall alliance decision-making, the changes in relevant indexes under the overall alliance decision-making are obtained. For the optimal level of CSR effort about the supply chain members, the supplier, the manufacturer and the retailer are all improved. For the supply chain member’s profit and the total system profit, if the supplier, the manufacturer, and the retailer reach the profit incremental distribution agreement based on local alliance decision-making with cost sharing in advance, the optimal profit of the supplier, manufacturer, retailer, and supply chain system are all increased. For CSR goodwill, the expectation and variance are increased. Therefore, in the process of supply chain CSR management, the supplier, the manufacturer, and the retailer should reach an agreement on profit increment distribution and coordinate CSR management. Compared with the other three decision-making situations, the overall alliance decision-making is the optimal decision-making. The supply chain members should strive to achieve this situation to obtain higher profit.

## Contributions and Limitations

This paper makes contributions in many aspects: (1) this paper studies the CSR of the supplier, the manufacturer, and the retailer three-tier supply chain for the first time. (2) This paper studies the CSR of supply chain under the action of external factors in a dynamic framework. (3) This paper considers the strategic cooperation of supply chain members, which makes the game closer to the reality.

Although this paper has some innovations in many aspects, it also has some limitations: (1) this paper only considers the CSR coordination in a single three-tier supply chain, and the CSR coordination of multiple three-tier supply chains can be further studied in the future.(2) The model assumptions are ideal. In the future, factors such as price, advertising, and after-sales service can be taken as endogenous variables to study their influence on supply chain CSR decision-making.

## Data Availability Statement

The original contributions presented in the study are included in the article/supplementary material, further inquiries can be directed to the corresponding author/s.

## Author Contributions

MY was responsible for conceptualization and methodology of the manuscript. ZY was responsible for software and investigation of the manuscript. YL was responsible for writing of the manuscript. XL was responsible for supervision, project administration, and validation of the manuscript. All authors contributed to the manuscript and approved the submitted version.

## Conflict of Interest

The authors declare that the research was conducted in the absence of any commercial or financial relationships that could be construed as a potential conflict of interest.

## Publisher’s Note

All claims expressed in this article are solely those of the authors and do not necessarily represent those of their affiliated organizations, or those of the publisher, the editors and the reviewers. Any product that may be evaluated in this article, or claim that may be made by its manufacturer, is not guaranteed or endorsed by the publisher.
